# What can dissaving tell us about catastrophic costs? Linear and logistic regression analysis of the relationship between patient costs and financial coping strategies adopted by tuberculosis patients in Bangladesh, Tanzania and Bangalore, India

**DOI:** 10.1186/s12913-015-1138-z

**Published:** 2015-10-22

**Authors:** Jason Madan, Knut Lönnroth, Samia Laokri, Stephen Bertel Squire

**Affiliations:** Division of Health Sciences, Warwick Medical School, Coventry, UK; Collaboration for Applied Health Research and Delivery, Liverpool School of Tropical Medicine and Warwick Medical School, Liverpool and Coventry, UK; Global TB Programme, World Health Organisation, Geneva, Switzerland; Department of Public Health Sciences, Karolinska Institutet, Stockholm, Sweden; Department of Global Health Systems and Development, School of Public Health and Tropical Medicine, Tulane University, New Orleans Louisiana, USA; Hoover Fellow of the Belgian American Educational Fund (BAEF), Brussels, Belgium; Department of Clinical Sciences, Liverpool School of Tropical Medicine, Liverpool, UK

**Keywords:** Monitoring, Evaluation, Assets, Loans, Dissaving, Catastrophic costs

## Abstract

**Background:**

Tuberculosis (TB) is a major global public health problem which affects poorest individuals the worst. A high proportion of patients incur ‘catastrophic costs’ which have been shown to result in severe financial hardship and adverse health outcomes. Data on catastrophic cost incidence is not routinely collected, and current definitions of this indicator involve several practical and conceptual barriers to doing so. We analysed data from TB programmes in India (Bangalore), Bangladesh and Tanzania to determine whether dissaving (the sale of assets or uptake of loans) is a useful indicator of financial hardship.

**Methods:**

Data were obtained from prior studies of TB patient costs in Bangladesh (*N* = 96), Tanzania (*N* = 94) and Bangalore (*N* = 891). These data were analysed using logistic and linear multivariate regression to determine the association between costs (absolute and relative to income) and both the presence of dissaving and the amounts dissaved.

**Results:**

After adjusting for covariates such as age, sex and rural/urban location, we found a significant positive association between the occurrence of dissaving and total costs incurred in Tanzania and Bangalore. We further found that, for patients in Bangalore an increase in dissaving of $10 USD was associated with an increase in the cost-income ratio of 0.10 (*p* < 0.001). For low-income patients in Bangladesh, an increase in dissaving of $10 USD was associated with an increase in total costs of $7 USD (*p* <0.001).

**Conclusions:**

Dissaving is potentially a convenient proxy for catastrophic costs that does not require usage of complex patient cost questionnaires. It also offers an informative indicator of financial hardship in its own right, and could therefore play an important role as an indicator to monitor and evaluate the impact of financial protection and service delivery interventions in reducing hardship and facilitating universal health coverage. Further research is required to understand the patterns and types of dissaving that have the strongest relationship with financial hardship and clinical outcomes in order to move toward evidence-based policy making.

## Background

Health care utilisation can lead to severe financial hardship, particularly for poorer households in low and middle income countries (LMICs). Hardship can arise even when care is ‘free’, due to direct costs such as transport and medication, and indirect costs (loss of income) [1]. Over 100 million individuals are pushed into poverty annually by health costs [2]. Reducing healthcare-related impoverishment, through improved design of healthcare services and access to financial protection [3], is essential for the progressive realization of universal health coverage [4]. The term ‘catastrophic health expenditure’ is commonly used to refer to direct healthcare costs that place excessive burdens on households. Health expenditure is said to be catastrophic if it ‘threatens a household’s ability to meet its subsistence needs’ [5–7]. Some have argued for a complementary indicator including direct non-medical costs and income, as these can be at least as problematic as direct medical costs [3, 8]. Catastrophic costs have been shown to be associated with adverse clinical outcomes in tuberculosis (TB) [9]. In recognition of the problem of costs faced by patients with TB [8, 10, 11], the World Health Organisation has adopted a post-2015 target of “no tuberculosis-affected household facing catastrophic costs due to tuberculosis” by 2020 [12].

Definition and measurement of catastrophic costs are important to support the evaluation and monitoring of both service delivery [13] and financial protection [14] interventions. Despite this, data on the financial burden of diseases such as TB impose on patients and their households, and the incidence of catastrophic costs, are not routinely collected. Unfortunately, standard definitions of catastrophic costs, based on cost-income ratios, create significant challenges for routine monitoring and evaluation. In practice, catastrophic costs are commonly defined as a threshold percentage of income or usual expenditure, as this relates costs to the financial capacity of a household. A range of thresholds have been used to denote catastrophic healthcare expenditure, such as >40 % of discretional income [15] or >10 % of total income [16]. There are no established thresholds for total costs, although a cut-off of >20 % of household income has been suggested specifically for people receiving care for tuberculosis, based on an association with poor treatment outcomes [9]. Calculation of patient costs involves the completion of questionnaires which may be cumbersome for routine use in monitoring and evaluation. Income may be challenging to assess for the most disadvantaged patient groups, who rely on a range of activities generating cash and in-kind income that is irregular and subject to seasonal fluctuation. An alternative ‘proxy means’ approach can be used in which living circumstances and possession of key items are used to classify socioeconomic status [17], but such measures can only provide an approximation to current household income.

Given these challenges, directly measurable proxy indicators of catastrophic costs would be beneficial. Low-income households often take out loans or sell assets to finance health-related expenditure [18]. This is sometimes referred to as ‘dissaving’, particularly in the economics literature, to highlight the fact that it reduces the financial strength of a household, just as saving increases a household’s resilience to financial shocks [19]. Assets are an important mechanism for saving in low income households, who may not have access to any type of financial institution, formal or informal [20]. Where households do have access to loans, the effects on household livelihoods can be severe and long-lasting [21]. For such households, dissaving is easier to measure than expenditure and income, and is likely to be associated with financial hardship. To explore the potential for using dissaving as a proxy indicator for catastrophic cost, we analysed the association between patient costs and dissaving for patients receiving tuberculosis treatment in India (Bangalore), Bangladesh and Tanzania.

## Methods

Data for Bangladesh and Tanzania were collected as a part of a study examining the potential financial impact of shorter TB treatment regimens [13]. Data on income, direct and indirect patient costs, asset sales, and loans were collected in 2011/2012 for 96 patients in Bangladesh and 94 patients in Tanzania, using a standard tuberculosis patient costing questionnaire [22]. Patients were selected to provide a representative sample of the population of adult TB patients in each country, using a sampling strategy described in detail in previous work [13]. Data were collected in the first 2 months and the last 2 months of treatment. Respondents were not asked their exact income; instead, they were asked in which of four pre-specified ranges their income lay. Additional questions on household structure, dwelling construction, and durable good ownership were used to compute Socioeconomic Scores (SES) based on statistics from Demographic and Household Surveys (DHS). Data for India were collected to assess costs incurred by all patients (*N* = 1106) newly registered for treatment in the city of Bangalore in 2005 under the Revised National tuberculosis Control Programme (RNTCP) [23]. Patients completed questionnaires, at the start and end of treatment, on direct and indirect costs, income, loans, and amounts raised through asset sales. SES were calculated using a standard-of-living instrument previously used in several studies of the socio-economic status of Indian tuberculosis patients. The costs included in both studies were-transportation from and to health facilities, medicines, food consumed during the visit, and facility fees. Data was also collected in Bangladesh and Tanzania on costs for food supplements taken on medical advice and accommodation (if required). These two items were not specifically included in the Bangalore questionnaire, although an option was included to provide ‘other’ costs incurred by patients. Cost and income data were measured in local currencies in each study and translated to United States Dollars (USD) assuming 45INR (Indian Rupees), 1570TZS (Tanzanian Shillings) and 80BDT (Bangladeshi Taka) per USD. Further details on the methods and the results from these studies have been reported elsewhere [13, 23, 24]. As this was a retrospective study, ethics approval was not required.

We reanalysed these patient cost datasets to explore the determinants of high or extreme costs and the relationship between costs and dissaving. SBS and KL were actively involved in the studies which collected these datasets, and granted access to JM to permit this re-analysis. We examined the distribution of costs relative to the median in each study, to identify potential drivers of high costs. We chose median costs as a convenient benchmark for the typical costs faced by participants in each study location. We chose to label costs ‘low’ if below the median value, ‘medium’ if greater than median but less than double the median, ‘high’ if greater than double median costs but less than triple, and ‘extreme’ if greater than triple median costs. These cut-off levels, though arbitrary, allow us to explore the extent of atypically high costs in a comparable way across locations. We carried out logistic and linear regression analyses to determine the relationship between costs and the presence and amount of dissaving. For the Bangalore dataset, we carried out additional regression analyses of the relationship between the level of dissaving and the cost/income ratio, to assess the coincidence of dissaving with ratio-based definitions of catastrophic cost. For the Bangladesh and Tanzania datasets, we performed separate regression analyses of the cost-dissaving relationship for those whose reported income is low, high or missing, as exact incomes were not reported. The explanatory variables considered in the analyses were cost, SES, location, age, gender and hospitalisation. All statistical analyses were carried out in R version 3.0.1.

## Results

Mean patient costs were considerably higher than median costs for all locations (40 % higher for Bangladesh and Tanzania, 85 % higher in Bangalore), reflecting a skewed distribution commonly found for costs. The results of our cost stratification are presented in Tables 1, 2 and 3. Cost data were provided by 891 (81 %) participants in Bangalore, and all participants in Bangladesh and Tanzania. The proportion of patients experiencing ‘extreme’ costs varied across locations - 18 % in Bangalore (*n* = 157), 10 % in Tanzania (*n* = 9), and 3 % in Bangladesh (*n* = 3). Patients experiencing extreme costs in Bangalore were more likely to be male (*p* < 0.01), and were more likely to report hospitalisation (*p* < 0.01), medium/high SES (*p* < 0.01) and dissaving (*p* < 0.01). Given the low numbers experiencing extreme costs in the other two sites, a more instructive comparison is between those who experience costs greater than 200 % of the median (i.e. ‘high’ or ‘extreme’ costs), and those who do not. The proportion experiencing ‘high’ or ‘extreme’ costs was 18 % in Bangladesh (*N* = 17) and 22 % in Tanzania (*N* = 21). In Tanzania, costs greater than 200 % of the median were significantly associated with hospitalisation (*p* < 0.01), whereas in Bangladesh, they was significantly associated with income in the higher band (*p* = 0.03).

**Table 1:** Information on dissaving and patient characteristics for Bangladesh tuberculosis patients, stratified by cost band

Cost band	Definition	No.	Mean patient cost (BDT)	% Rural	Mean age	% Male	Mean SES score	No. reporting high income	No. hospitalised	No. dissaving			
										Loan	Sale	Both	All
Low	<100 % of median	48	4400	71 %	42	58 %	−0.097	9 (19 %)	1 (2 %)	18 (38 %)	2 (4 %)	4 (8 %)	24 (50 %)
Medium	100–200 % of median	33	12,100	74 %	42	76 %	−0.085	6 (18 %)	3 (9 %)	13 (39 %)	1 (3 %)	5 (15 %)	19 (58 %)
High	200–300 % of median	12	18,700	67 %	34	64 %	0.258	7 (58 %)	1 (7 %)	7 (58 %)	0 (0 %)	0 (0 %)	7 (58 %)
Extreme	>300 % of median	3	99,400	33 %	41	67 %	1.233	1 (33 %)	2 (67 %)	3 (100 %)	0 (0 %)	0 (0 %)	3 (100 %)
All	Any cost level	96	11,700	70 %	41	66 %	0.019	23 (24 %)	7 (7 %)	41 (43 %)	3 (3 %)	9 (9 %)	53 (55 %)

*BDT* Bangladesh Taka. Median Patient cost 8,200BDT. High Income defined as >1935BDT / week

**Table 2:** Information on dissaving and patient characteristics for Tanzania tuberculosis patients, stratified by cost band

Cost band	Definition	No.	Mean patient cost (000 s TZS)	% Rural	Mean age	% Male	Mean SES score	% Reporting high income	% Hospitalised	No. dissaving			
										Loan	Sale	Both	All
Low	<100 % of median	47	139	41 %	37	60 %	0.246	7 (15 %)	4 (9 %)	7 (15 %)	11 (23 %)	5 (11 %)	23 (49 %)
Medium	100–200 % of median	28	294	50 %	39	54 %	0.249	7 (25 %)	4 (14 %)	3 (11 %)	8 (29 %)	8 (29 %)	19 (68 %)
High	200–300 % of median	10	544	60 %	42	80 %	0.042	3 (30 %)	3 (30 %)	0 (0 %)	1 (10 %)	5 (50 %)	6 (60 %)
Extreme	>300 % of median	9	1053	22 %	44	67 %	0.204	5 (55 %)	7 (78 %)	3 (33 %)	3 (33 %)	3 (33 %)	9 (100 %)
All	Any cost level	94	323	44 %	39	66 %	0.221	22 (23 %)	18 (19 %)	13 (14 %)	23 (24 %)	21 (22 %)	57 (61 %)

*TZS* Tanzania Shilling. Median Patient cost 230,000TZS. High Income defined as >20,000TZS / week

**Table 3:** Information on dissaving and patient characteristics for Bangalore tuberculosis patients, stratified by cost band

Cost band	Definition	No.	Mean patient cost (INR)	Mean income (INR / yr)	Mean age	% Male	% Low SES	% Hospitalised	No. dissaving			
									Loan	Sale	Both	All
Low	<100 % of median	445	1700	4900	33	51 %	51 %	20 %	69 (16 %)	6 (1 %)	8 (2 %)	83 (19 %)
Medium	100–200 % of median	191	5800	5700	35	59 %	51 %	48 %	73 (38 %)	6 (3 %)	9 (5 %)	88 (46 %)
High	200–300 % of median	98	9700	5400	35	63 %	49 %	65 %	45 (46 %)	3 (3 %)	8 (8 %)	56 (57 %)
Extreme	>300 % of median	157	24,000	5800	35	74 %	38 %	78 %	79 (50 %)	3 (2 %)	23 (15 %)	105 (67 %)
All	Any cost level	891	7400	5300	35	59 %		42 %	266 (30 %)	18 (2 %)	48 (5 %)	332 (37 %)

*INR* Indian Rupee. Median patient cost 4000INR

Dissaving rates overall were comparable between Tanzania and Bangladesh (61 % vs 55 %), and lower in Bangalore(37 %). This difference was largely due to dissaving rates in the low cost band, which were lower in Bangalore (19 %, vs 50 % in Bangladesh and 49 % in Tanzania). Only 6 % of dissavers in Bangladesh and 5 % in Bangalore relied solely on asset sales to raise funds. In Bangladesh, assets were only sold where costs were low or medium (i.e. less than 200 % of the median), whereas there was no clear relationship between costs and type of dissaving in Bangalore. Assets sales were more prevalent in Tanzania, where 40 % of dissavers did not take out loans. The difference in mean income between those reporting ‘low’ versus ‘extreme’ costs in the Bangalore dataset was only 20 % (Table 3), and the relationship between income and cost band was not statistically significant (*p* = 0.13). Income levels were reported by 62 % of respondents in Tanzania and 67 % of respondents in Bangladesh. Although the questionnaires offered respondents a choice of 4 income bands, only four respondents in Bangladesh and six respondents in Tanzania reported incomes in the higher two bands. We therefore aggregated the higher three levels in the stratification analysis, dividing respondents into low and high income, using the upper limit of the lowest income band in the questionnaires as the threshold (1935 BDT per week in Bangladesh and 20,000TZS per week in Tanzania). Higher income was associated with cost level in both countries at the 10 % significance level (Bangladesh *p* = 0.08, Tanzania *p* = 0.07).

Results from logistic regressions to predict the probability of dissaving are given in Table 4. In Tanzania, dissaving was significantly positively associated with cost (*p* = 0.03) and male gender (*p* = 0.005). In Bangladesh, these variables were also positively associated with dissaving, but not significantly. No other variables were significantly associated with dissaving in either location. We carried out a similar analysis for Bangalore, with predictors similar except for the lack of rural patients, the availability of income data, and the characterisation of SES as a 3-level factor. We found significant positive associations between the presence of dissaving and cost (*p* = 0.005), hospitalisation (*p* < 0.001), and low socioeconomic level (*p* = 0.011). There was a significant negative association between dissaving and income (*p* < 0.001). We then explored the association between patient cost and the amount of dissaving among the subset of patients (57 in Tanzania, 53 in Bangladesh and 332 in Bangalore) who reported dissaving at any amount (Table 5). We found a significant positive relationship between costs and dissaving, after allowing for factors such as income, socioeconomic status and hospitalisation, for Bangladesh (*p* < 0.001) and Bangalore (*p* < 0.001). The relationship for Tanzania was positive but not significant (*p* = 0.201).

**Table 4:** Coefficients for logistic regression models predicting likelihood of dissaving of any amount

	Mean (se) coefficient values		
Predictors	Tanzania (*N* = 96)	Bangladesh (*N* = 94)	Bangalore (*N* = 891)
Intercept	2.370e + 00 (1.268e + 00)	1.793e + 00 (1.473e + 00)	−1.220e + 00 (4.378e-01)**
Total cost	2.929e-06 (1.347e-06)*	4.001e-05 (3.687e-05)	7.844e-05 (1.192e-05)***
Age	−2.922e-02 (1.956e-02)	−2.239e-02 (1.393e-02)	−1.192e-04 (2.895e-05)
Female gender	−1.552e + 00 (5.479e-01)**	−6.049e-01 (4.677e-01)	−7.655e-03 (5.494e-03)
Hospitalisation	−6.382e-01 (7.796e-01)	−1.095e + 00 (1.175e + 00)	7.995e-01 (1.684e-01)***
Rural location	−3.299e-01 (5.091e-01)	6.742e-01 (8.317e-01)	NA
SES	−1.068e + 00 (6.869e-01)	−7.520e-01 (6.295e-01)	NA
Low SES	NA	NA	8.800e-01 (3.488e-01)*
Medium SES	NA	NA	4.240e-01 (3.371e-01)
Income	NA	NA	−1.192e-04 (2.895e-05)***

‘***’*p* = 0.001 ‘**’*p* = 0.01 ‘*’p = 0.05

**Table 5:** Coefficients for linear regression models predicting total costs for patients who dissave

	Mean (se) coefficient values		
Predictors	Tanzania *N* = 57	Bangladesh *N* = 53	Bangalore *N* = 332
Intercept	3.043e + 05 (1.630e + 05)	1.889e + 04 (8.133e + 03)*	1.036e + 04 (2.977e + 03)***
Dissaving amount	2.181e-01 (1.684e-01)	6.779e-01 (6.212e-02)***	7.648e-01 (6.057e-02)***
Age	−7.726e + 01 (1.313e + 02)	−7.726e + 01 (1.313e + 02)	−7.041e + 01 (4.033e + 01)
Female gender	8.589e + 03 (4.410e + 03)	8.589e + 03 (4.410e + 03)	2.815e + 03 (1.126e + 03)*
Hospitalisation	−3.896e + 05 (8.473e + 04)***	−1.431e + 04 (6.368e + 03)*	3.201e + 03 (1.140e + 03)**
Rural location	1.609e + 05 (7.900e + 04)*	−7.219e + 03 (7.410e + 03)	NA
SES	−2.195e + 05 (1.251e + 05)	1.056e + 04 (5.116e + 03)*	NA
Low SES	NA	NA	−8.996e + 03 (2.431e + 03)***
Medium SES	NA	NA	−8.679e + 03 (2.382e + 03)***
Income	NA	NA	5.759e-01 (2.089e-01)**

‘***’*p* = 0.001 ‘**’*p* = 0.01 ‘*’p = 0.05

Of the 891 Bangalore respondents reporting cost and income, 762 (86 %) reported costs greater than 20 % of annual income, and 661 (74 %) reported costs greater than 40 % of total annual income. There was a significant positive relationship between dissaving and the ratio of cost to income, after allowing for age, gender, socioeconomic status and reported hospitalisation (Table 6). An increase in dissaving of $10 was associated with an increase in the cost-income ratio of 0.10 (*p* < 0.001). Table 7 presents the results from cost regressions stratified by income band in Bangladesh and Tanzania. Results are presented for those participants who provided their income band (Table 7a), and for all participants (Table 7b). The latter were calculated by imputing missing income bands from other participant characteristics (age, gender, location, SES and hospitalisation). Among Bangladeshi low-income dissavers, each $10 increase in dissaving was associated with higher costs of $7 (*p* < 0.001). There was no statistically significant relationship between the level of dissaving and patient costs among high-income Bangladeshis, high-income Tanzanians, or low-income Tanzanians. These results were robust to the method used for analysis in the presence of missing data (complete case analysis or imputation of income bands).

**Table 6:** Coefficients for linear regression model predicting cost-income ratios for Bangalore patients who dissaved

Predictors	Mean (se) coefficient values
Intercept	1.263e + 00 (9.024e-01)
Dissaving amount	2.279e-04 (2.176e-05)***
Age	2.025e-04 (1.454e-02)
Female gender	5.483e-01 (4.056e-01)
Hospitalisation	9.420e-01 (4.113e-01)*
Low SES	−1.914e-01 (8.065e-01)
Medium SES	−1.051e + 00 (8.267e-01)

‘***’*p* = 0.001 ‘*’p = 0.05

**Table 7:** Linear regression model coefficients predicting total patient cost by income band for those who dissaved

a: Complete case analysis								
	Mean (se) coefficient values							
	Tanzania				Bangladesh			
Predictors	Income < 20,000TZS / week		Income > 20,000TZS / week		Income < 1935 BDT / week		Income > 1935 BDT / week	
	*N* = 23		*N* = 16		*N* = 25		*N* = 11	
Intercept	1.376e + 05	(1.435e + 05)	−1.466e + 05	(1.047e + 06)	1.170e + 04	(1.373e + 04)	2.591e + 04	(1.075e + 04)
Dissaving amount	9.507e-02	(1.565e-01)	4.619e-01	(5.965e-01)	7.073e-01	(8.668e-02)***	2.763e-02	(1.192e-01)
Age	1.600e + 03	(3.048e + 03)	1.664e + 04	(1.736e + 04)	−3.163e + 01	(2.281e + 02)	6.530e + 01	(3.257e + 02)
Female gender	−3.683e + 04	(2.074e + 05)	1.517e + 05	(3.533e + 05)	1.823e + 04	(1.136e + 04)	NA	
Hospitalisation	NA		−2.868e + 05	(3.977e + 05)	−1.362e + 04	(9.631e + 03)	−3.507e + 03	(8.264e + 03)
Rural location	3.749e + 04	(8.546e + 04)	2.933e + 05	(2.893e + 05)	7.289e + 03	(1.265e + 04)	−3.016e + 04	(7.867e + 03)*
SES	1.879e + 04	(1.743e + 05)	−4.369e + 05	(2.798e + 05)	3.940e + 03	(8.415e + 03)	1.728e + 04	(6.022e + 03)*
b: Analysis with imputation of missing income band from participant characteristics (age, gender, location, SES and hospitalisation)								
	Mean (se) coefficient values							
	Tanzania				Bangladesh			
Predictors	Income < 20,000TZS / week		Income > 20,000TZS / week		Income < 1935 BDT / week		Income > 1935 BDT / week	
	*N* = 35		*N* = 23		*N* = 41		*N* = 12	
Intercept	4.020e + 05	2.804e + 05	−6.513e + 05	5.874e + 05	9.205e + 03	(8.980e + 03)	2.091e + 04	(1.130e + 04)
Dissaving amount	7.359e-02	1.889e-01	4.792e-01	3.782e-01	7.634e-01	(6.476e-02)***	3.834e-02	(1.314e-01)
Age	1.152e + 03	3.386e + 03	1.712e + 04	9.020e + 03	−1.810e + 01	(1.384e + 02)	2.778e + 02	(3.248e + 02)
Female gender	−6.014e + 04	1.152e + 05	4.260e + 04	1.946e + 05	7.789e + 03	(4.779e + 03)	NA	
Hospitalisation	NA		3.338e + 05	1.782e + 05	−1.107e + 04	(6.978e + 03)	−7.406e + 03	(8.675e + 03)
Rural location	−6.015e + 04	1.032e + 05	1.902e + 05	1.857e + 05	2.243e + 03	(8.193e + 03)	−2.783e + 04	(8.520e + 03)*
SES	−7.030e + 04	1.883e + 05	−3.359e + 05	2.035e + 05	4.261e + 03	(5.506e + 03)	1.805e + 04	(6.626e + 03)*

‘***’*p* = 0.001 ‘*’p = 0.05

## Discussion

The measurement of costs and income involves numerous challenges. Dissaving is, by comparison, easier to measure, indicates by definition a financial weakening of a household, and is a widely-used coping strategy [18]. It is therefore a potential proxy for catastrophic costs in routine monitoring and evaluation. We analyse the link between dissaving, costs, income, and catastrophic costs, using data collected in three countries across two continents,. The first part of our analysis explores the link between extreme cost and several potential explanatory variables, including dissaving. Our motivation was that cost distributions are commonly ‘fat-tailed’, reflecting high prevalence of ‘extreme’ costs likely to lead to financial hardship. We use median cost as a yardstick to identify extreme costs as it is simple to calculate and reflects costs typically faced by others in the same location. Hospitalisation was associated with extreme costs in Bangalore, and with high/extreme costs in Tanzania, Bangladesh, which had the lowest rate of hospitalisation, also had the lowest prevalence of extreme costs. This suggests that that service delivery models minimising hospitalisation should be prioritised, and financial protection offered when hospitalisation is unavoidable, which is consistent with previous findings that inefficient patient management can be a major cause of catastrophic health expenditure [1]. Income was not associated with extreme costs in Bangalore or high/extreme costs in Tanzania. Even in Bangladesh, where there was an association between high/extreme cost and income band, two of the three respondents reporting extreme costs also reported low income. Dissaving (at any level) was associated with extreme costs in Bangalore, but the relationship between dissaving and cost band was not significant in other locations. This may be due to the sample size, but might also be related to the fact that dissaving was more prevalent in Bangladesh (53 %) and Tanzania (61 %) than in Bangalore (37 %). Monitoring whether or not TB patients dissave is less likely to be sufficient in locations where it is commonplace.

There are two concerns with benchmarking against median costs as a tool for monitoring and evaluation. Firstly, interventions to reduce median costs will reduce the absolute value of thresholds based on it. A solution to this might be to benchmark against a stable measure such as a pre-defined context-specific measure of costs reflecting what is tolerable by poor households. Secondly, this does not take income, and hence ability to pay, into account. Our analysis suggests this may not be a major concern, since income is not strongly associated with cost, although this may not be true in every setting. All 157 respondents reporting extreme costs in Bangalore also reported a cost/income ratio greater than 40 %. Households facing ‘extreme’ costs are therefore largely a subset of those facing catastrophic costs; identifying and addressing drivers of ‘extreme’ costs (other than income variation) can therefore be seen as a stepping-stone towards the goal of eliminating catastrophic costs entirely.

We found consistent evidence of associations between cost-income ratios and both the presence and level of dissaving. In Bangalore, the probability of dissaving increased with costs and decreased with income, and there was a strong association between the amount of dissaving and the cost-income ratio, which supports using the former as a proxy for catastrophic costs. Evidence from Bangladesh and Tanzania was less conclusive, largely because these were smaller studies, and collected income data as bands rather than actual amounts. Even so, there were several statistically significant associations consistent with a relationship between dissaving and catastrophic cost, and none inconsistent with such a relationship. However, the sample size and lack of precise values for income are limitations, as is the fact that none of the studies whose data we used were explicitly designed to evaluate the use of dissaving as a proxy for catastrophic costs. In particular, data from Bangladesh and Tanzania are not available for the first 2 months of the continuation phase (months 3 and 4). This is more likely to be a significant issue for calculating dissaving than costs, as little is known about the timing of asset sales and uptake of loans, whereas it is more reasonable to assume that costs are spread evenly throughout the continuation phase. Given these limitations, further research is required to validate the use of dissaving as an indicator of catastrophic costs, and to explore the timing of dissaving relative to the incurring of costs. Further evidence is also required on how universally dissaving measures could be applied – in some settings, for example, there may be cultural barriers to the take-up or reporting of loans.

Our analysis suggests that dissaving can be a useful indicator in the evaluation and monitoring of financial protection and service delivery interventions aimed at reducing catastrophic costs. Further evidence would inform how this indicator is used – we expect it will supplement other measures of financial distress where available. Such measures could be used in combination to identify catastrophic costs, for example by comparing the amount of dissaving to the total amount of healthcare expenditure, using empirically determined thresholds. Dissaving may also capture financial distress that is not reflected in cost-income ratios, for example when households face multiple illnesses that are catastrophic together but not singly [6].

Our results also illustrate different strategies for dissaving – dissavers in Tanzania, for example, were more likely to sell assets than in Bangladesh or Bangalore. It is likely that the nature of dissaving will influence its relationship to financial hardship. It may therefore be useful to distinguish between types of dissaving when using this measure as a signal for financial distress. For example, it may be possible to distinguish between ‘planned dissaving’ and ‘distressed dissaving’. The former would involve measures designed for protecting households from financial shocks (e.g. cash savings, jewellery, or low-interest loans). The latter would involve dissaving actions that households adopt due to a lack of alternatives, despite significant immediate consequences for household well-being. Those who take out high-interest loans, or sell assets that generated a high proportion of household income, for example, would seem to be at greatest risk of failing to meet basic subsistence needs as a result. A further aim for future research would be to explore the relationship between the type of dissaving and the consequences for household wellbeing, in order to enhance the value of dissaving measures as an indicator of catastrophic cost.

## Conclusion

Diseases such as TB, which disproportionately affect poor households, can have a devastating effect on the financial security of those households. The costs incurred in relation to TB have been shown to appear early in the care-seeking pathway, and this has a direct impact on patient health, as it restricts the ability to access and adhere to subsequent care [3, 22]. Dissaving is an informative indicator of financial hardship, and a convenient potential proxy for catastrophic costs. It does not require usage of complex patient questionnaires, making it a particularly suitable indicator for routine monitoring and evaluation. It is likely that instruments to assess dissaving could be used across a wide range of illnesses with minimal alteration, whereas patient cost questionnaires require substantial adaptation to reflect specific care-seeking and treatment pathways. Further research is required to understand the patterns and types of dissaving that have the strongest relationship with financial hardship.

## Abbreviations

BDT: Bangladeshi taka
DHS: Demographic and household surveys
INR: Indian rupees
LMICs: Low and middle income countries
RNTCP: Revised national tuberculosis programme
SES: Socioeconomic scores
TZS: Tanzanian shillings
TB: Tuberculosis
USD: United States dollars
